# Effects of the selective inhibition of proteasome caspase-like activity by CLi a derivative of nor-cerpegin in dystrophic mdx mice

**DOI:** 10.1371/journal.pone.0215821

**Published:** 2019-04-23

**Authors:** Yeranuhi Hovhannisyan, Gagik Melikyan, Nathalie Mougenot, Jacqueline Gao-Li, Bertrand Friguet, Denise Paulin, Zhenlin Li, Arnaud Ferry, Onnik Agbulut

**Affiliations:** 1 Sorbonne Université, Institut de Biologie Paris-Seine (IBPS), CNRS UMR 8256, Inserm ERL U1164, Biological Adaptation and Ageing, Paris, France; 2 Yerevan State University, Department of Organic Chemistry, Yerevan, Armenia; 3 Sorbonne Université, Plateforme PECMV, UMS28, Paris, France; 4 Sorbonne Université, Centre de Recherche en Myologie, Institut de Myologie, INSERM U974, Paris, France; 5 Sorbonne Paris Cité, Université Paris Descartes, Paris, France; University of California, Davis, UNITED STATES

## Abstract

Previous studies have shown that proteasome inhibition can have beneficial effects in dystrophic mouse models. In this study, we have investigated the effects of a new selective proteasome inhibitor, CLi, a strong caspase-like inhibitor of the 20S proteasome, on skeletal and cardiac muscle functions of mdx mice. In the first series of experiments, five-month-old male mdx mice (n = 34) were treated with 2 different doses (20 and 100 μg/kg) of CLi and in the second series of experiments, five-month-old female mdx (n = 19) and wild-type (n = 24) mice were treated with 20 μg/kg CLi and Velcade (1 mg/kg) for 1-month. All animals were treadmill exercised twice a week to worsen the dystrophic features. In the first series of experiments, our results demonstrated that 20 μg/kg CLi did not significantly increase absolute and specific maximal forces in skeletal muscle from male mdx mice. Moreover, the higher susceptibility to contraction induced skeletal muscle injury was worsened by 100 μg/kg CLi since the force drop following lengthening contractions was increased with this high dose. Furthermore, we found no differences in the mRNA levels of the molecular markers implicated in dystrophic features. Concerning cardiac function, CLi had no effect on left ventricular function since ejection and shortening fractions were unchanged in male mdx mice. Similarly, CLi did not modify the expression of genes implicated in cardiac remodeling. In the second series of experiments, our results demonstrated an improvement in absolute and specific maximal forces by CLi, whereas Velcade only increased specific maximal force in female mdx mice. In addition, exercise tolerance was not improved by CLi. Taken together, our results show that CLi treatment can only improve maximal force production in exercised female mdx mice without affecting either exercice tolerance capacity or cardiac function. In conclusion, selective inhibition of caspase-like activity of proteasome with CLi has no compelling beneficial effect in dystrophic mdx mice.

## Introduction

Duchenne muscular dystrophy is a fatal genetic disorder affecting both skeletal and cardiac muscles. It is caused by dystrophin deficiency. Dystrophin is a subsarcolemmal protein, thought to play a role in force transmission, sarcolemma stability, localization and function of different proteins that trigger damage process in its absence. The mdx mouse is a widely used mouse model for Duchenne muscular dystrophy, it exhibits muscle weakness, i.e. reduced specific maximal force (absolute maximal force generated relative to muscle cross-sectional area or weight) [1–5] as well as muscle degeneration and regeneration. The muscle of Mdx mice is also more susceptible to damage caused by lengthening (eccentric) contractions. Thus, less than 10 lengthening contractions cause an immediate marked force drop in mdx mice but not in wild-type mice [6–9]. In addition to these skeletal muscle dystrophic features, the heart of mdx mice also exhibits signs of cardiomyopathy that are more pronounced in old as compared to young mdx mice [10,11].

Over the last years, pharmacological interventions modulating catabolic pathways have been investigated in the mdx mouse. Several of these pre-clinical studies have reported that proteasome inhibition rescues the expression of the dystrophin glycoprotein complex, and improves histological dystrophic features in skeletal muscle of mdx mice [12,13]. Moreover, it was also shown that proteasome inhibition in dystrophin deficient mice rescued the level and function of the cardiac voltage-gated sodium channel that plays an important role in cardiac excitability [14]. A recent study by Selsby et al showed that proteasome inhibition with MG-132 failed to improve muscle weakness and fragility [15]. Whereas, in contrast, Bonuccelli et al demonstrated that administration of the proteasomal inhibitor MG-132 reduces muscle membrane damage and effectively rescues the expression levels and plasma membrane localization of dystrophin, ß-dystroglycan, α-dystroglycan, and α-sarcoglycan in skeletal muscle fibers from mdx mice [16]. In spite of these contradictory observations, both the impact of long-term proteasome inhibition and the impact of selective inhibition of proteasome peptidase activities on dystrophic muscle function have not yet been established. In the present study, we aimed to examine whether selective pharmacological proteasome inhibition would improve both skeletal muscle and heart functions in mdx mice.

We recently demonstrated that CLi (1,1-dimethyl furo[4-benzylamino]pyridine-3-one), a derivative of nor-cerpegin, is a strong caspase-like inhibitor of the 20S constitutive proteasome [17]. Among a set of 18 nor-cerpegin C^1^ and C^4^ derviatives tested by Hovhanissyan et al [17], CLi, bearing a benzylamino group at position C^4^, strongly and selectively inhibited caspase-like activity of the 20S proteasome. *In vitro* studies using purified proteasome and HeLa cells demonstrated that the other proteasome activities were not modified by treatment with CLi [17]. In order to explore the impact of CLi treatment in mdx mice, the mice were treated for 1 month with CLi. In addition, the treated mdx mice were exercised twice a week on a motorized treadmill to increase dystrophic features in hindlimb muscle, since the extent of dystrinopathology is relatively mild in mdx mice [18,19]. Following this protocol, we demonstrated that selective inhibition of 20S proteasome caspase-like activity can slightly improve maximal force production in exercised mdx mice without affecting either exercice tolerance capacity or cardiac function.

## Materials & methods

### Animals

All procedures were performed in accordance with national and European legislations, in conformity with the Public Health Service Policy on Human Care and Use of Laboratory Animals under the license 75–1102. All animal studies were approved by our institutional Ethics Committee “Charles Darwin” (Permit number: 01361.03) and conducted according to the French and European laws, directives, and regulations on animal care (European Commission Directive 86/609/EEC). All surgery was performed under sodium pentobarbital anesthesia, and all efforts were made to minimize suffering. To confirm the selective proteasome inhibition in mdx mice, the mice were treated during 1 week and 1 hour after the last injection the muscles were removed to mesure proteasome peptidase activities. Then, two series of experiments were carried out. In the first series of experiments, thirty-four 5-month-old male mdx mice were used. They were distributed into the following groups: sedentary and treated with vehicle (Sed, n = 7), exercised and treated with vehicle (Run, n = 9), exercised and treated with 20 μg/kg CLi (Run+CLi-Low, n = 9) and exercised and treated with 100 μg/kg CLi (Run+CLi-High, n = 9). In the second series of experiments, nineteen 5-month-old female mdx mice were used. They were distributed in the following groups: sedentary and treated with vehicle (Sed, n = 4), exercised and treated with vehicle (Run, n = 5), exercised and treated with 20 μg/kg CLi (Run+CLi-Low, n = 5) and exercised and treated with 1 mg/kg Velcade (Run+Velcade, n = 5). In addition, twenty-four wild-type C57BL6J mice were used as control. CLi (MW 268 g/mol) and Velcade (MW 384 g/mol) were dissolved in DMSO at 75 mM and 130 mM, respectively and then diluted in PBS to obtain the final concentration. The animals were treated 3 times a week (i.p.) for CLi and 2 times a week (i.p.) for Velcade. Mice were exercised for 4 weeks on a horizontal motorized treadmill, 2x30 min/week (20 cm/s) since this protocol has been shown to increase dystrophic features in previous studies [18,19]. Moreover, this exercise protocol is recommended for pre-clinical studies (Treat-NMD, DMD_M.2.1.001).

### Exercise tolerance measurement

Two days after the last injection, a treadmill running test was performed to measure the exercise tolerance of the mice [19]. Exercise tolerance testing consisted of a run to exhaustion. The speed was increased from 5 to 20 cm/s during the first 2 min, then the speed was maintained constant during the next 10 min, and the speed was increased by 5 cm/s every 10 min until exhaustion. Exhaustion was defined as when the mouse was no longer able to maintain its normal running position and/or showed frequent contact with the shock grid (≤ 0.2 mA) at the rear of the treadmill.

### Muscle force measurement

Two days after the running test and four days after the last treatment, maximal tetanic isometric force and susceptibility to contraction-induced injury (see below) were evaluated by measuring the *in situ* tibialis anterior (TA) muscle contraction in response to nerve stimulation, as described previously [20]. Briefly, mice were anesthetized using pentobarbital (60 mg/kg i.p.). After body temperature was maintained at 37°C using radiant heat, the knee and foot were fixed with pins and clamps and the distal tendon of the muscle was attached to a lever arm of a servomotor system (305B, Dual-Mode Lever, Aurora Scientific, Aurora, Canada) using a silk ligature. The sciatic nerve was proximally crushed and distally stimulated by a bipolar silver electrode using supramaximal square wave pulses of 0.1 ms duration (10 V). We measured the absolute maximal force that was generated during isometric tetanic contractions in response to electrical stimulation (125 Hz, 500ms). Absolute maximal force was determined at L0 (length at which maximal tension was obtained during the tetanus). Absolute maximal force was normalized to the muscle weight as an estimate of specific maximal force.

Susceptibility to contraction-induced injury was estimated from the force drop resulting from lengthening contraction-induced injury. The sciatic nerve was stimulated for 700ms (frequency of 125Hz). A maximal isometric contraction of the TA muscle was initiated during the first 500ms. Then, muscle lengthening (10% L0) at a velocity of 5.5 mm/s (0.85 fiber length/s) was imposed during the last 200 ms. Nine lengthening contractions of the muscle were performed, each separated by a 60 s rest period. All contractions were made at an initial length L0. Maximal isometric force was measured 60 s after each lengthening contraction and expressed as a percentage of the initial maximal force. Data was acquired with a sampling rate of 100 kHz (Powerlab 4/25, ADInstrument, Oxford, United Kingdom). After contractile measurements, the animals were killed by cervical dislocation and muscles were weighed and stored for further analyses.

### Echocardiography

Echocardiography was performed on lightly anesthetized mice under isoflurane (induction with 2% isoflurane-100% O_2_, and maintained with 0.2% isoflurance-100% O_2_). Non-invasive measurements of left ventricular dimensions were evaluated using echocardiography-Doppler (GE Medical System Co, Vivid 7 Dimension/Vivid7 PRO) with an ultrasound probe frequency range from 9 to 14 MHz, as previously described [8]. Two-dimensional guided time-motion recording mode (parasternal long-axis view) was used to measure the left ventricular (LV) end-diastolic and -systolic diameters (LVEDD and LVESD, respectively), LV intraventricular septal and posterior wall thicknesses (IVS and LVPW, respectively) and heart rate. All measurements were made online on freeze frame images in triplicate and averaged by an investigator blinded to the treatment group. LV fractional shortening (FS) was calculated using the formula: (LVEDD-LVESD)/LVEDD×100. LV end-diastolic and -systolic volumes (EDV and ESV, respectively) were calculated using a half-ellipsoid model of the LV. From these volumes, LV ejection fraction (EF) was calculated using the formula: (EDV-ESV)/EDVx100.

### Relative quantification of gene expression by qPCR

Total RNA was extracted from the apex of hearts and tibialis anterior muscles using TRIzol Reagent (Thermo Fisher Scientific, Saint-Herblain, France) following the manufacturer’s instructions. From 250 ng of extracted RNA, the first-strand cDNA was then synthesized using the RevertAid First Strand cDNA Synthesis Kit (Thermo Fisher Scientific, Saint-Herblain, France) with anchored-oligo(dT)18 primer and according to the manufacturer's instructions. Using the Light Cycler 480 system (Roche Diagnostics), the reaction was carried out in duplicate for each sample in a 6 μl reaction volume containing 3 μl of SYBR Green Master Mix, 500 nM of the forward and reverse primers each and 3 μl of diluted (1:25) cDNA. The thermal profile for SYBR Green qPCR was 95°C for 8 min, followed by 40 cycles at 95°C for 15 s, 60°C for 15 s and 72°C for 30 s. To exclude PCR products amplified from genomic DNA, primers were designed, when possible, to span one exon-exon junction. The mean gene expression stability of 2 genes, *Sdha* (Succinate deshydrogenase complex, subunit A, flavoprotein), *Hmbs* (Hydroxymethylbilane synthase), was used as the reference transcript. Data were collected and analyzed using the LightCycler 480 software release 1.5.0 (Roche Diagnostics). Primers sequences used in this study are available on request.

### Proteasome peptidase activities measurement

Proteasome containing muscle homogenates were prepared just after dissection using ice-cold homogenization buffer containing: 20 mM Tris–HCl (pH 7.6), 250 mM NaCl, 3 mM EDTA, 3 mM EGTA and 2 mM DTT. Samples were minced with scissors and homogenised using plastic pestles, incubated 30 min on ice, then centrifuged at 12,000 g for 15 min at 4°C. Protein concentration was measured using the Bradford method with bovine serum albumin as a standard. The proteasomal chymotrypsin-like, trypsin-like and caspase-like activities of the 20S catalytic core were assayed using the fluorogenic substrates *N*-Succinyl-Leu-Leu-Val-Tyr-7-amino-4-methylcoumarin (Suc-LLVY-AMC, Enzo Life Sciences, Villeurbanne, France), Bz-Val-Gly-Arg-7-amino-4-methylcoumarin (Bz-VGR-AMC, Enzo Life Sciences, Villeurbanne, France) and Z-Leu-Leu-Glu-7-amino-4-methylcoumarin (Z-LLE-AMC, Enzo Life Sciences, Villeurbanne, France), respectively. The mixture, containing 10 μg of total protein in 20 mM Tris (pH 8) and 10% gylcerol, was incubated at 37°C with 20 μM peptide substrates in a final volume of 100 μl. Enzymatic kinetics were monitored in a temperature-controlled microplate fluorimetric reader. Excitation/emission wavelengths were 350/440 nm. The difference between assays with or without MG-132, a proteasome inhibitor, represented the proteasome peptidase specific activity.

### Statistical analysis

Groups were generally statistically compared using one way-analysis of variance. The factor was generally the group (Sed, Run, Run+CLi-Low, Run+CLi-High or Sed, Run, Run+CLi-Low, Run+Velcade). If appropriate, subsequent post-hoc analysis (Bonferroni) was performed. For groups that did not pass tests of normality and equal variance, non-parametric tests were used (Kruskal Wallis and Wilcoxon). A *p* value < 0.05 was considered significant. Values are means ± SEM.

## Results

### Skeletal muscle function

**Fig 1:**
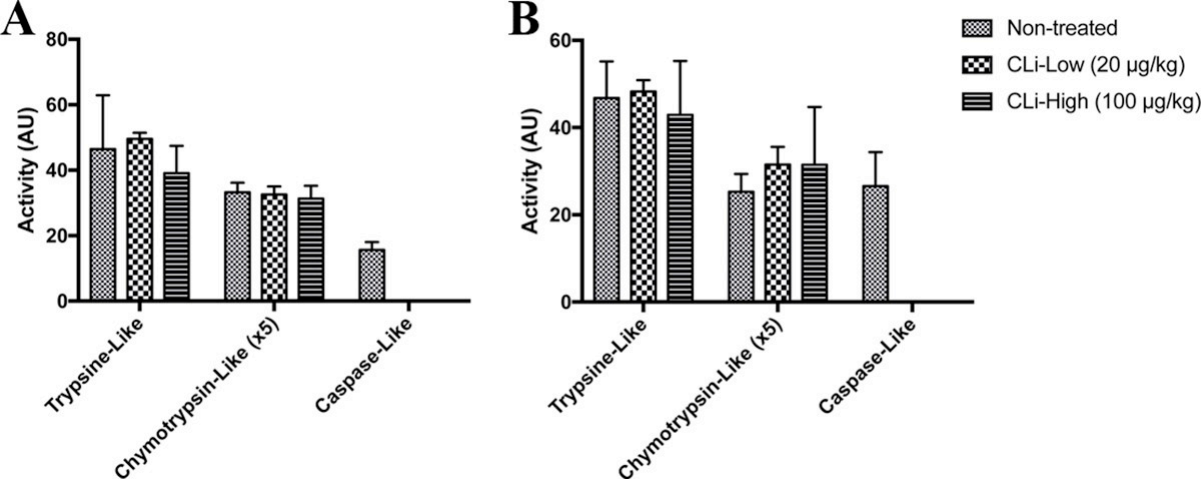
Proteasome peptidase activities in treated male and female mdx mice. Proteasomal chymotrypsin-like, trypsin-like and caspase-like activities of the 20S catalytic core were measured using the fluorogenic substrates in treated male (A) and female (B) mice tibialis anterior muscle. CLi-Low: CLi treated mdx mice with 20 μg/kg; CLi-High: CLi treated mdx mice with 100 μg/kg. Note the selective inhibition of caspase-like activity. n = 3–4 per group.

To confirm the selectivity of this new proteasome inhibitor in mdx mice, CLi, a strong caspase-like 20S proteasome inhibitor was injected intraperitonealy in mdx mice. Animals were treated with CLi at two different doses, i.e. low dose 20 μg/kg or high dose 100 μg/kg. Proteasomal chymotrypsin-like, trypsin-like and caspase-like activities of the 20S catalytic core were measured using the fluorogenic substrates, after 1 week of treatment. We found that both 20 μg/kg and 100 μg/kg CLi treatments selectively inhibit caspase-like activity of 20S proteasome in skeletal muscle (Fig 1) (*p*<0.05).

**Fig 2:**
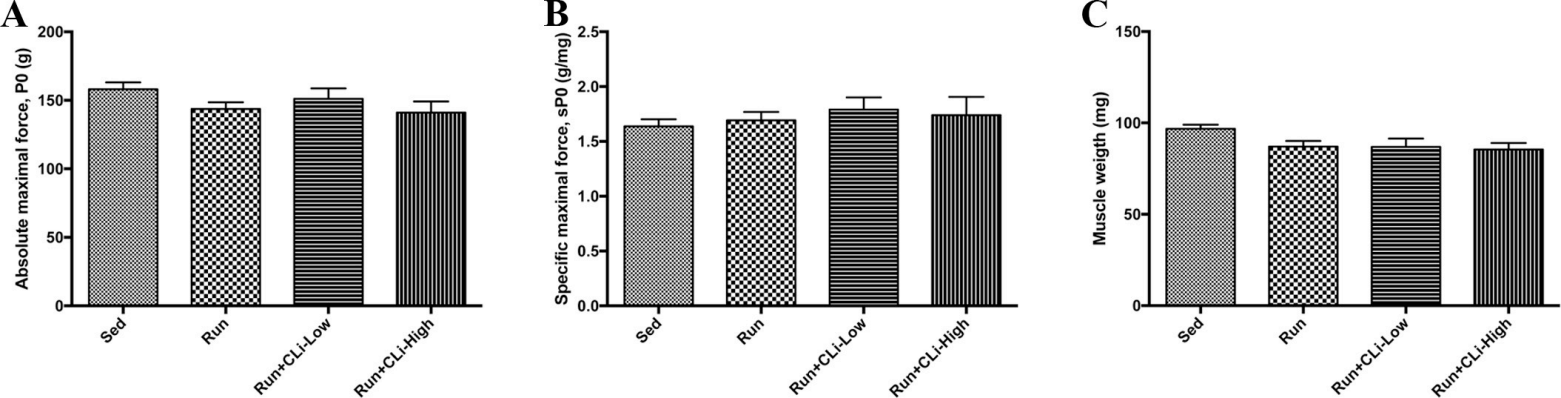
Maximal force in treated mdx mice in the first series of experiments. A) Absolute maximale force (Po); B) Specific maximal force (sPo) C) Muscle weight; of tibialis anterior muscles of mdx mice. Sed: non-treated sedentary mdx mice; Run: exercised and treated with vehicle; Run+Cli-Low: exercised and treated mdx mice with 20 μg/kg CLi; Run+CLi-High: exercised and treated mdx mice with 100 μg/kg CLi. n = 14–18 per group.

**Fig 3:**
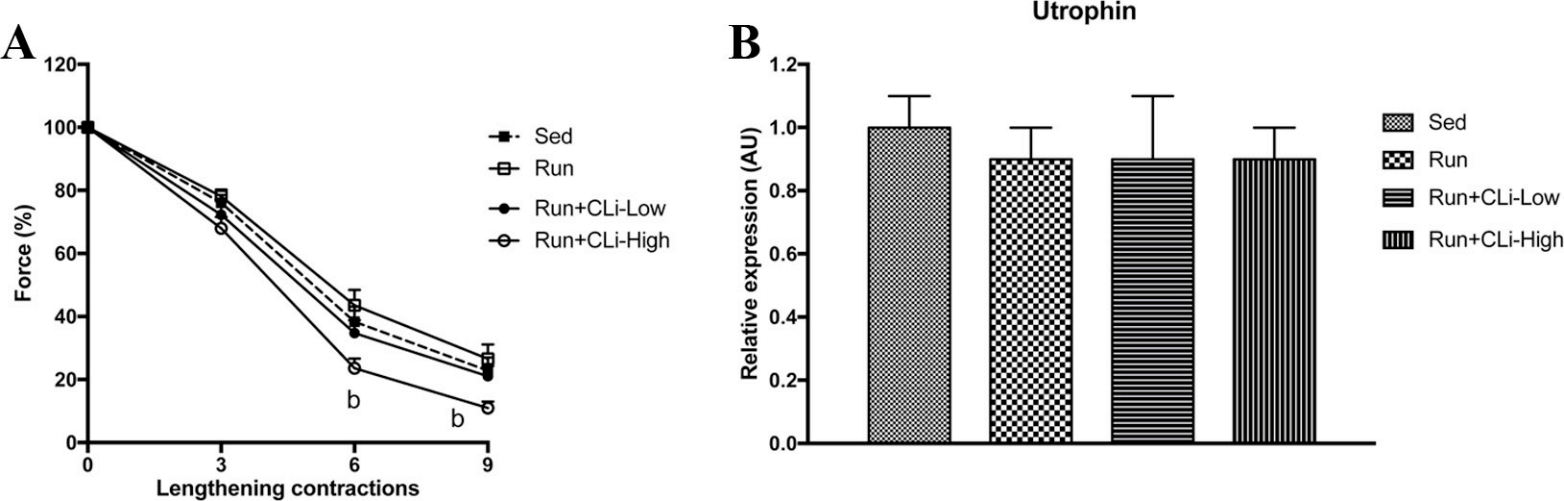
**Muscle susceptibility to lengthening contractions (fragility) in the first series of experiments (A) and utrophin expression (B) in treated mdx mice.** Sed: non-treated sedentary mdx mice; Run: exercised and treated with vehicle; Run+CLi-Low: exercised and treated mdx mice with 20 μg/kg CLi; Run+CLi-High: exercised and treated mdx mice with 100 μg/kg CLi. n = 14–18 per group for A; n = 7–9 per group for figure B. b: significantly different from Run mice (*p*<0.05).

**Fig 4:**
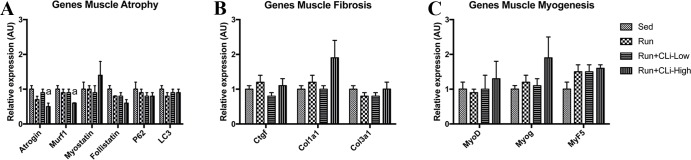
**Relative quantification by real-time PCR of genes involved in muscle atrophy (A), muscle fibrosis (B) and myogenesis (C) in treated mdx mice in the first series of experiments.** Sed: non-treated sedentary mdx mice; Run: exercised and treated with vehicle; Run+CLi-Low: exercised and treated mdx mice with 20 μg/kg CLi; Run+CLi-High: exercised and treated mdx mice with 100 μg/kg CLi. Col1a1, collagen type 1 alpha 1; Col3a1, collagen type 3 alpha 1; Ctgf, connective tissue growth factor; Myog, myogenin. n = 7–9 per group. a: significantly different from Sed mice (p<0.05).

In the first series of experiments, CLi was injected 3 times a week in five-month old male mdx mice for 1 month. In order to worsen the dystrophic features, mdx mice were treadmill exercised twice a week. One month after treatment (four days after the last treatment), *in situ* force production in response to nerve stimulation was measured to determine the effects of CLi treatment on dystrophic features of mdx mice. We found that absolute maximal force and specific maximal force, which is the normalization of absolute maximal force by muscle weight, were not significantly increased in Run+CLi-low mdx as compared to Run mdx (Fig 2A and 2B) (*p*>0.05), indicating that muscle weakness of mdx mice was not significantly improved by the CLi treatment. Moreover, there was no difference in muscle weights between the different mdx groups (Fig 2C) (*p*>0.05). The immediate force drop following lengthening contractions, is widely used to measure the extent of muscle damage caused by contraction in mdx mice, i.e., fragility, another important functional dystrophic features with weakness. We found that the force drops following the 6^th^ and 9^th^ lengthening contractions were greater in high dose CLi treated mice (Run+CLi-High) as compared to non-treated (Run) mice (*p*<0.05) (Fig 3A), indicating that a high dose of CLi increases the susceptibility to lengthening contractions. As demonstrated in Fig 3B, the greater force drops in high dose CLi treated mice was not related to a reduced level of utrophin mRNA, a dystrophin analog (Fig 3B) (*p*>0.05). Moreover, qPCR analyses revealed no difference of several molecular dystrophic features between different groups, i.e. muscle atrophy (Atrogin, murf1, myostatin, follistatin, P62 and LC3), fibrosis (connective tissue growth factor, collagen type 1 alpha 1, collagen type 3 alpha 1) and myogenesis (MyoD, Myogenin and Myf5), underlying again the inefficacy of CLi treatment in male mdx mice (Fig 4A–4C) (*p*>0.05).

**Fig 5:**
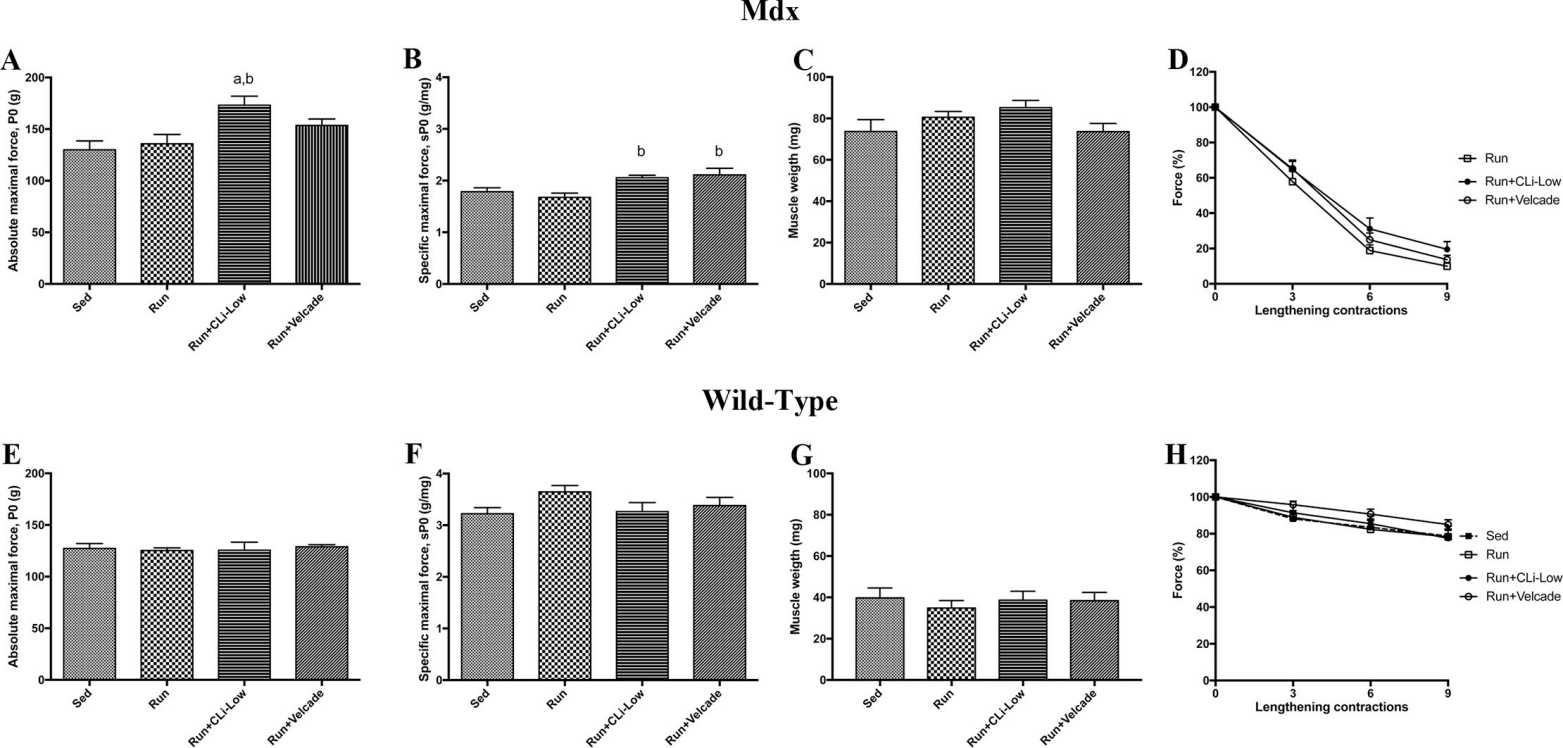
**Muscle tetanic isometric force and muscle susceptibility to lengthening contractions (fragility) in treated mdx (A-D) and wild-type (E-H) mice in the second series of experiments.** A and E) Absolute maximal force (Po); B and F) Specific maximal force (sPo), C and G) Muscle weight; of tibialis anterior muscles of mdx mice; D and H) Muscle susceptibility to lengthening contractions. Sed: non-treated sedentary mdx mice; Run: exercised and treated with vehicle; Run+CLi-Low: exercised and treated mdx mice with 20 μg/kg CLi; Run+Velcade: exercised and treated mdx mice with 800 μg/kg Velcade. n = 8–10 per group. a: significantly different from Sed mice (p<0.05), b: significantly different from Run mice (p<0.05).

In a second series of experiments, we used five-month old female mdx mice, to study the effect of low dose CLi treatment, that was sufficient to fully inhibit caspase-like activity of 20S proteasome, without any detrimental effect on fragility. Low dose CLi was also compared to the classical proteasome inhibitor, Velcade. Interestingly, in the female mdx mice, as demonstrated in Fig 5A, absolute maximal force was improved in the CLi-Low treated (Run+CLi-Low) mdx mice as compared to the Velcade-treated and Run mdx mice (Fig 5A) (*p*<0.05). Moreover, specific maximal force was slightly improved both in CLi-Low-treated and Velcade-treated mdx mice as compared to Run mdx mice (Fig 5B), demonstrating that these two treatments improve muscle weakness in female mdx mice. Concerning muscle weight, we did not find any significant differences between different mdx groups (Fig 5C). As demonstrated in Fig 5D, neither CLi-Low treatement nor Velcade treatment worsens the susceptibility to lengthening contractions in mdx mice, confirming the first series of experiments. The experiments realized with wild-type mice did not demonstrated any adverse effects of CLi and Velcade treatments on maximal force and muscle susceptibility to lengthening contractions (fragility) (Fig 5E–5H). Moreover, qPCR analyses revealed no difference in molecular markers of muscle atrophy, fibrosis, and myogenesis (data not shown). Taken together, selective inhibition of caspase-like activity of the constitutive proteasome using CLi-Low can have beneficial effects on skeletal muscle function in dystrophic mdx mice, at least in female mdx mice.

### Cardiac function

**Fig 6:**
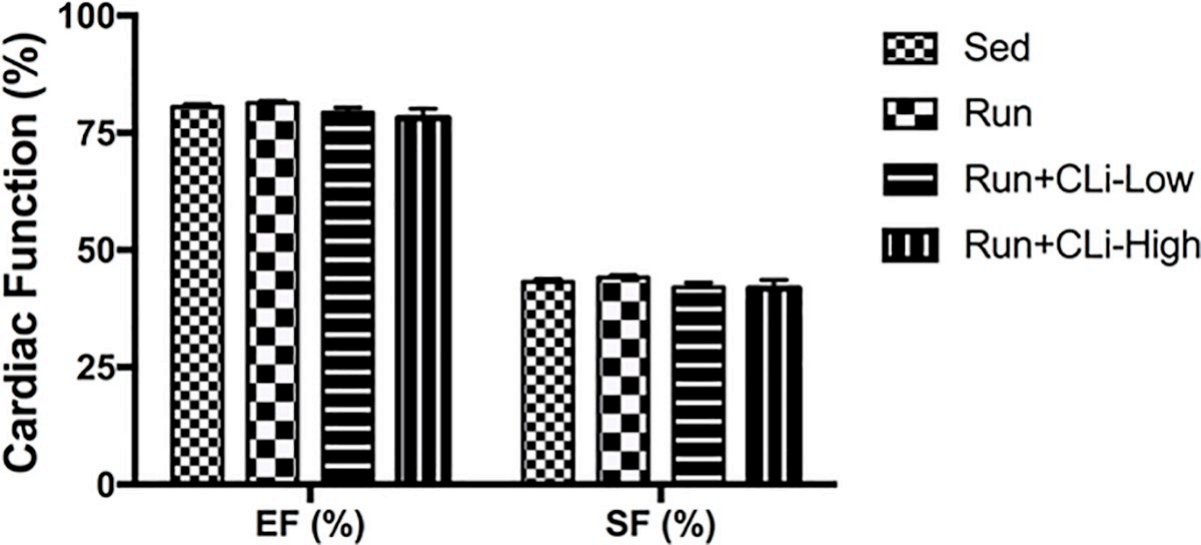
Cardiac function in treated mdx mice in the first series of experiments. Left ventricular ejection fraction (EF); Left ventricular shortening fraction (FS). Sed: non-treated sedentary mdx mice; Run: exercised and treated with vehicle; Run+CLi-Low: exercised and treated mdx mice with 20 μg/kg CLi; Run+CLi-High: exercised and treated mdx mice with 100 μg/kg CLi. n = 7–9 per group.

**Fig 7:**
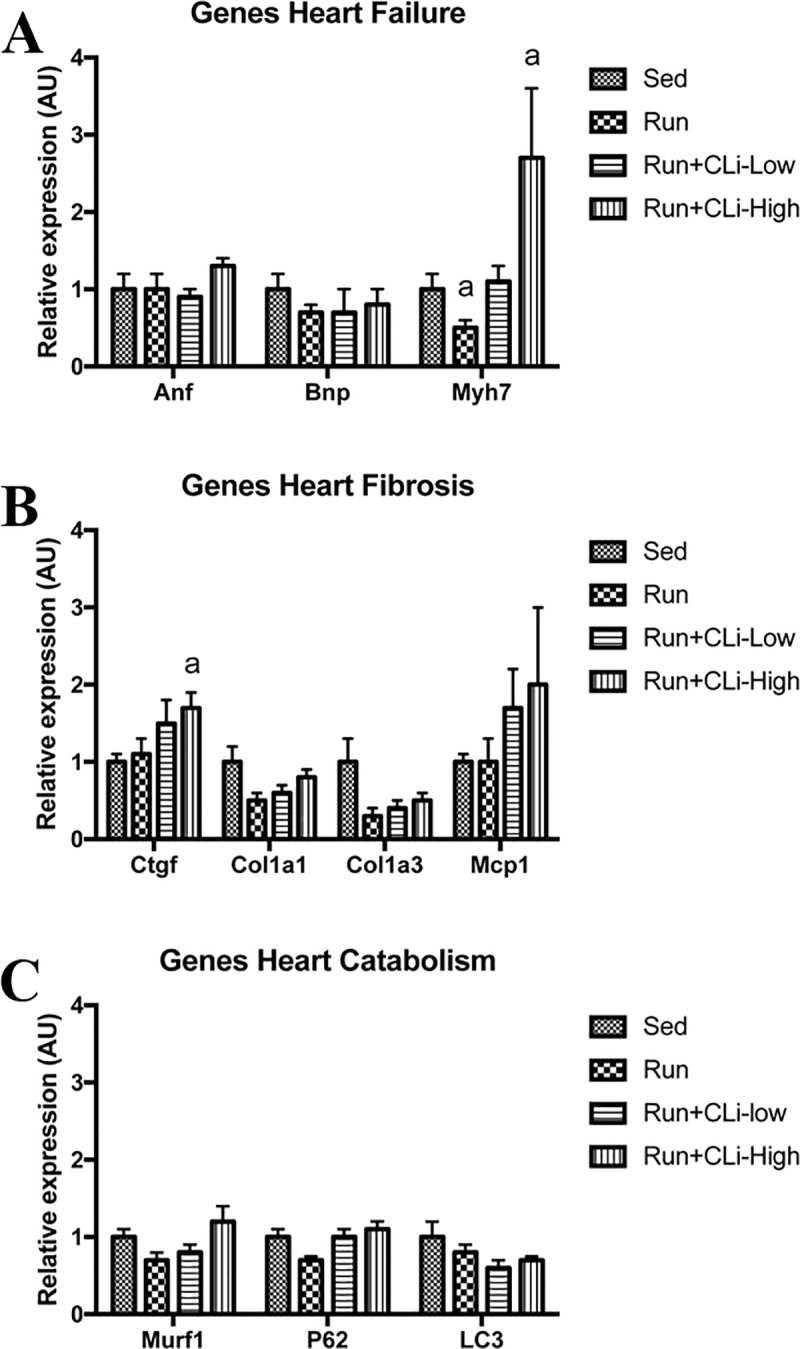
**Relative expression of genes involved in cardiac fibrosis (A) and remodeling (B) and catabolism (C) in treated mdx mice in the first series of experiments.** Sed: non-treated sedentary mdx mice; Run: exercised and treated with vehicle; Run+CLi-Low: exercised and treated mdx mice with 20 μg/kg CLi; Run+CLi-High: exercised and treated mdx mice with 100 μg/kg CLi. Anf, atrial natriuretic factor; Bnp, brain natriuretic peptide; Col1a1, collagen type 1 alpha 1; Col3a1, collagen type 3 alpha 1; Ctgf, connective tissue growth factor; Mcp1, monocyte chemoattractant protein-1; Myh7, myosin heavy chain 7. n = 7–9 per group. a: significantly different from Sed mice (p<0.05).

In order to evaluate the impact of CLi treatments on heart function of dystrophic mdx, we first performed echocardiographic measurements in treated mice in the first series of experiments. As shown in Fig 6, echocardiographic analysis revealed that ejection fraction (EF) and shortening fraction (FS) did not differ between groups, indicating that the CLi treatment has no effect on left ventricular function (*p*>0.05). Moreover, we found globally no marked changes in mRNA levels of different genes involved in heart failure (Anf, Bnp, Myh7), fibrosis (connective tissue growth factor, collagen type 1 alpha 1, collagen type 3 alpha 1, monocyte chemoattractant protein-1) or cardiac catabolism (Murf1, P62, LC3) (Fig 7A–7C), except the increased expression of connective tissue growth factor (Ctgf) and Myh7 in the high dose CLi treated (Run+CLi-High) mdx mice as compared to non-treated mdx mice (Fig 7B) (*p*<0.05). Concerning the second series of experiments, qPCR analyses revealed again no difference in the mRNA levels of different genes involved in fibrosis and in cardiac remodeling (data not shown).

**Fig 8:**
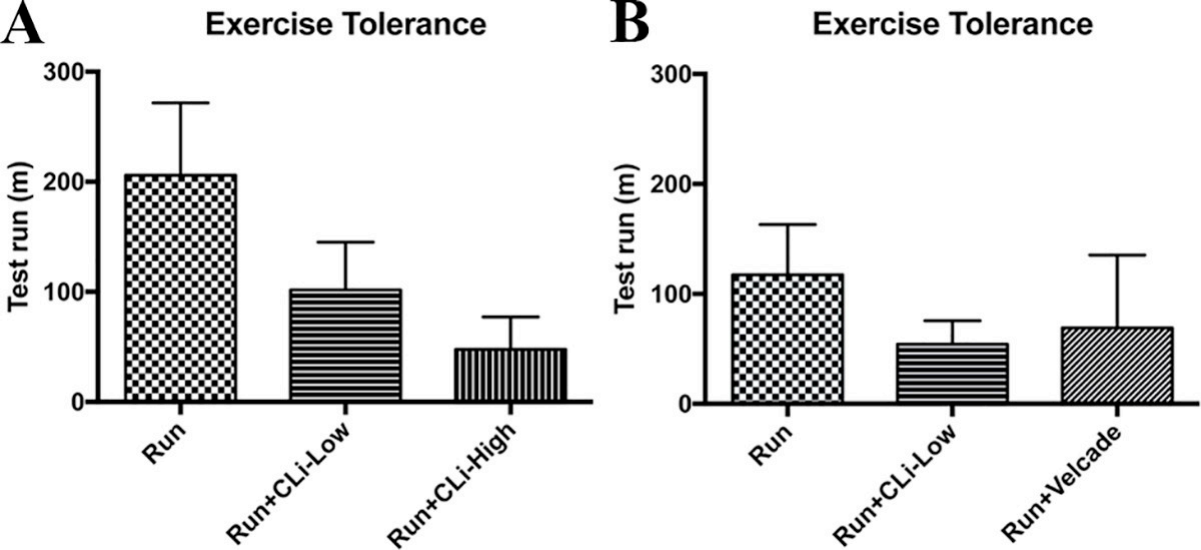
Exercise tolerance (distance to exhaustion) in mdx mice in the first and second series of experiments. Sed: non-treated sedentary mdx mice; Run: exercised and treated with vehicle; Run+CLi-Low: exercised and treated mdx mice with 20 μg/kg CLi; Run+CLi-High: exercised and treated mdx mice with 100 μg/kg CLi; Run+Velcade: exercised and treated mdx mice with 800 μg/kg Velcade. n = 7–9 per group.

In addition, we evaluated the potential effects of CLi treatment on exercise tolerance of mdx mice in the first and second series of experiment. As shown in Fig 8, both CLi and Velcade treatments tend to decrease the exercise tolerance capacity of mdx mice but the differences failed to reach a significant level. Taken together, neither CLi nor Velcade treatment had beneficial effects on cardiac function and exercise tolerance in mdx mice.

## Discussion

Mdx mice exhibit increased proteasome activity and proteasome inhibition has been previously shown to rescue the expression of the dystrophin glycoprotein complex, to improve histological dystrophic features in skeletal muscle and to restore cardiac sodium current in dystrophin deficient mice [12–14,16,21–22]. Moreover, exercise increases proteasome activity in both skeletal and cardiac muscles, at least in healthy mice [23,24]. Therefore, in the present study we sought to determine whether inhibition of the 20S proteasome caspase-like activity by CLi would be beneficial for skeletal and cardiac muscle functions in exercised dystrophic mdx mice. CLi (1,1-dimethyl furo[4-benzylamino]pyridine-3-one), a derivative of nor-cerpegin, is a strong caspase-like inhibitor of the 20S constitutive proteasome [17]. Our results demonstrate that both 20 μg/kg and 100 μg/kg CLi treatments inhibit selectively the caspase-like activity of the 20S proteasome in skeletal muscle. One-month after treatment, our results showed that inhibition of the 20S proteasome caspase-like activity with the selective nor-cerpegin family proteasome inhibitor slightly improved maximal force production in exercised mdx mice without affecting either exercice tolerance capacity or cardiac function. In conclusion, selective inhibition of caspase-like activity of the proteasome with CLi has no compelling beneficial effect in dystrophic mdx mice.

However, we did observe that maximal force production was increased by CLi-Low, at least in exercised female mdx mice, indicating that CLi can potentially improve muscle weakness in dystrophic muscle, as previously shown for velcade. However, we demonstrate that fragility, i.e., the susceptibility to contraction-induced muscle damage was worse in 100 μg/kg CLi treated mdx mice. These results completed and extended those of a recent study showing that the effect of an 8 day treatment with MG-132 increases the fragility in sedentary mdx mice [15]. The increased fragility induced by CLi treatment was not explained by a reduced expression of utrophin. However, it remains to be determined whether CLi treatment could reduce utrophin at the protein level. Also, it is very unlikely that CLi would induced dystrophin expression, as expected from previous study with proteasome inhibition [16]. Since the increased fragility in mdx mice is related to defective muscle excitability [25], it is likely that the high CLi dose further deteriorates muscle excitability. Whatever the underlying mechanism, our result suggests that CLi treatment can increase fragility and thus accelerates skeletal muscle damage, i.e. the dystrophic process including repeated cycles of degeneration-regeneration. As demonstrated in this study and also by others [15], the functional dystrophic features can be worsened with proteasome inhibition whereas histological features were attenuated [13,16], highlighting the importance of evaluating the effectiveness of a pre-clinical treatment on the basis of a physiological approach.

Another novel finding of the present study was that CLi treament did not modify cardiac function in exercised mdx mice. In fact, ejection and shortening fractions were not affected by the CLi treatments. A limitation of the present study was that left ventricular function was intact in our 5-month old mdx mice [8,10,11] and was not worsened by exercise (the present study). In this condition, our results demonstrate that CLi treatment does not induce any detrimental effect on cardiac function. Therefore, it remains to be determined if CLi treatment have a beneficial effect on a deficient cardiac function in mdx mice. This point needs to be examined in future studies using recently developped and more relevant rat models of DMD [26,27]. Moreover, since CLi also possibly reduces exercise tolerance in mdx mice, it will be important to test this aspect.

In both series of experiments a recommended treadmill running protocol was performed in an attempt to induce muscle injury and to accentuate dystrophic features [19]. This exercise protocol is recommended for pre-clinical studies (Treat-NMD, DMD_M.2.1.001). However, we found that muscle weakness and fragility were not exacerbated in the Run mdx as compared to Sed mdx mice, confirming a previous study [28]. These results indicate that the use of a more severe murine Duchenne muscular dystrophy model than the exercised mdx mice would be more useful in pre-clinical studies.

In conclusion, our results demonstrate that inhibition of the 20S proteasome caspase-like activity with the selective nor-cerpegin family proteasome inhibitors present some slight functional beneficial effects in adult exercised mdx mice. However, neither exercise tolerance nor cardiac function were improved by CLi. Together, the fact that CLi treatment present only a limited beneficial effect on skeletal muscle suggests that it has no potential to be a pharmacological therapy for Duchenne muscular dystrophy. Moreover, this study underlines the importance of the evaluation of physiological parameters rather than morphological characterization to evaluate therapeutic potential of drug candidates for Duchenne muscular dystrophy.
